# 2056 Culture meets person-centered care: An empirical study of mental health and care planning among Latinx and Asian communities

**DOI:** 10.1017/cts.2018.245

**Published:** 2018-11-21

**Authors:** Miraj U. Desai, Nadika Paranamana, Maria Restrepo-Toro, Luz Ocasio, Yolanda Herring, Merarilisse Crespo, Larry Davidson

**Affiliations:** 1 Yale School of Medicine; 2 University of Hartford

## Abstract

OBJECTIVES/SPECIFIC AIMS: This poster will present preliminary results from a study examining whether person-centered care planning—a new innovation in community mental health care—responds to the culture of, and helps reduce health disparities among, Latinx and Asian populations. METHODS/STUDY POPULATION: The study was funded by an NIMH/NIH Administrative Supplement for Minority Health and Mental Health Disparities Research and approved by the Institutional Review Board of the authors’ university. Participants included 26 mental health clients and 12 mental health providers of diverse backgrounds. The study employed empirical qualitative methods to explore client understandings of mental health, client experiences of culture and discrimination, and the process of care engagement and care planning from both client and provider perspectives. The analysis team itself included people of Latinx and Asian background, as well as a person with lived experience of mental health recovery. RESULTS/ANTICIPATED RESULTS: We anticipate that the results will show ways in which person-centered care successfully incorporates clients’ goals, but that there will also be evidence of ways in which the clinical encounter struggles to incorporate more social, collective, and cultural values and approaches. DISCUSSION/SIGNIFICANCE OF IMPACT: The poster will present up-to-date findings on this project, which speaks to pressing issues of health equity and community engagement for 2 of the fastest growing populations in the country.

